# Schulische Gesundheitsförderung in pandemischen Zeiten. Ergebnisse der COVID-HL-Schulleitungsstudie

**DOI:** 10.1007/s00103-022-03535-w

**Published:** 2022-05-06

**Authors:** Kevin Dadaczynski, Orkan Okan, Melanie Messer

**Affiliations:** 1 Fachbereich für Pflege und Gesundheit, Hochschule Fulda, Leipziger Str. 123, 36037 Fulda, Deutschland; 2 Public Health Zentrum (PHZF), Hochschule Fulda, Fulda, Deutschland; 3 Zentrum für Angewandte Gesundheitswissenschaften, Leuphana Universität Lüneburg, Lüneburg, Deutschland; 4 Fakultät für Sport- und Gesundheitswissenschaften, Technische Universität München, München, Deutschland; 5 Abteilung Pflegewissenschaft II, Universität Trier, Trier, Deutschland

**Keywords:** Gesundheitsfördernde Schule, SARS-CoV‑2, Schüler*innen, Lehrkräfte, Schulleitungen, Health promoting school, SARS-CoV‑2, Pupils, Teachers, School principals

## Abstract

**Hintergrund:**

Die vorliegende Studie untersucht, in welchem Ausmaß Schulen Maßnahmen der Gesundheitsförderung und Prävention während der COVID-19-Pandemie umsetzen. Von besonderem Interesse sind hierbei Unterschiede nach demografischen Variablen, Schulform, Bundesland und die Beteiligung an Landesinitiativen der Gesundheitsförderung.

**Methodik:**

Im Rahmen des COVID-Health-Literacy-Netzwerks wurde von März bis April 2021 eine Onlinestudie mit 2186 Schulleitungen in Baden-Württemberg, Hessen, Niedersachsen und Nordrhein-Westfalen durchgeführt. Der Umsetzungsstand der COVID-19-bezogenen schulischen Gesundheitsförderung und Prävention wurde mittels eines eigenentwickelten Instruments untersucht. Nach Analyse der faktoriellen Struktur des Instruments erfolgten uni- und bivariate Auswertungen.

**Ergebnisse:**

Es lassen sich 3 Dimensionen des Umsetzungsstandes der schulischen Gesundheitsförderung identifizieren (1. COVID-19-bezogene Unterstützung der Schüler*innen, 2. Gesundheitsförderliche Gestaltung von Lehr‑, Lern- und Arbeitsbedingungen, 3. Prinzipien der Gesundheitsfördernden Schule). Eine geringe Umsetzung liegt für Aspekte der Lehr‑, Lern- und Arbeitsbedingungen sowie für Partizipation und die Kooperation mit schulexternen Akteuren vor. Signifikante Unterschiede des Umsetzungsstands ergeben sich zugunsten von weiblichen und älteren Schulleitungen sowie Grundschulen. Außerdem zeigen sich nicht homogene Unterschiede nach Bundesland. Differenziert nach Teilnahme an einem Landesprogramm findet sich lediglich für Schulen mit Zertifikat im Bereich Gesundheitsförderung ein höherer Umsetzungsstand.

**Diskussion:**

Die Ergebnisse geben Hinweise darauf, dass die COVID-19-Pandemie für Schulen ein disruptives Ereignis darstellt, welches die Umsetzung schulischer Gesundheitsförderung erschwert. Vor allem gesundheitsförderliche Arbeitsbedingungen, Partizipation und Kooperation sollten in den Fokus genommen werden.

**Zusatzmaterial online:**

Zusätzliche Informationen sind in der Online-Version dieses Artikels (10.1007/s00103-022-03535-w) enthalten

## Abstract (en)

**Background:**

This study examines the extent to which schools implement activities on health promotion and prevention during the COVID-19 pandemic. Moreover, potential differences with regard to demographic variables, school type, state, and participation in state health promotion initiatives are determined.

**Methods:**

As part of the international COVID Health Literacy Network, an online-based study was conducted from March to April 2021 with 2186 school principals from the German federal states of Baden-Württemberg, Hesse, Lower Saxony, and North Rhine-Westphalia. The implementation status of COVID-19-related school health promotion was assessed using a self-developed instrument. After examining the factorial structure of the instrument, univariate and bivariate data analyses were performed.

**Results:**

Three dimensions of implementing school health promotion can be identified: (1) COVID-19-related support for pupils, (2) health promoting design of teaching, learning, and working conditions, and (3) the principles of Health Promoting Schools. A low level of implementation is observed for aspects of teaching, learning, and working conditions as well as for participation and cooperation with community stakeholders. Significant differences are observed, with female, older, and primary school principals reporting a higher implementation status, while for federal states non-homogenous differences are found. Stratified by participation in state health promotion initiatives, only schools with a certificate in health promotion show a higher level of implementation.

**Discussion:**

The results indicate that the COVID-19 pandemic is a disruptive event for schools, impeding the implementation of holistic activities on health promotion and prevention. In particular, more attention should be given to the creation of health promoting working conditions, participation, and community cooperation.

## Einleitung

Die COVID-19-Pandemie stellt das Schulsystem in Deutschland in vielerlei Hinsicht vor große Herausforderungen. Mit den im März 2020 durch die Kultusministerien der Bundesländer umgesetzten ersten Schulschließungen traten die Umstellung von Präsenzunterricht auf digitalen Fernunterricht (Homeschooling) und die Einrichtung einer Notbetreuung für Kinder und Jugendliche von Eltern aus systemrelevanten Berufen in Kraft. Die schrittweise Öffnung des Schulbetriebs war begleitet von zahlreichen Maßnahmen zur Verhinderung der Verbreitung des Coronavirus (z. B. schulindividuelle Hygienepläne, entzerrte und verkleinerte Klassen, Wechselunterricht). Während der Start des Schuljahres 2020/2021 unter neuen, verschärften Hygienevorgaben erfolgte, führten steigende Infektionszahlen im Dezember 2020 zu einer erneuten Schließung der Schulen im gesamten Bundesgebiet. Sowohl die im Herbst und Winter 2021 dominierende Coronavirusvariante Delta als auch die seit Januar 2022 verbreitete Omikronvariante haben erneut Diskussionen um mögliche Schulschließungen entfacht.

Diese und weitere pandemiebedingte Einflüsse haben nicht nur zu zahlreichen Lerneinbußen und einer Verstärkung soziallagenbedingter Bildungsungleichheiten geführt [1–3], sondern sind auch aus Public-Health-Perspektive von besonderer Bedeutung. So liefern verschiedene empirische Befunde Hinweise auf negative gesundheitliche Auswirkungen der COVID-19-Pandemie, z. B. auf psychische Belastungen und die psychische Gesundheit [4–6], den Gewichtsstatus [6–8] oder auch die körperliche Aktivität [9, 10] von Kindern und Jugendlichen.

Auch für Lehrkräfte sind psychische Belastungen und Beanspruchungen dokumentiert, u. a. infolge von Mehrarbeit oder der Umstellung auf den Fernunterricht [11–14]. Erste Ergebnisse für Schulleitungen belegen zudem ein hohes Ausmaß von Arbeitsstress und gesundheitsriskantem Arbeitsbewältigungsverhalten, welches sich vor allem in einem hohen und auf Dauer belastenden Arbeitstempo sowie einer Ausdehnung der Arbeit (z. B. Erreichbarkeit in der Freizeit, Verzicht auf Erholungszeiten) bemerkbar macht [15].

Die Befunde machen deutlich, dass Gesundheit als Thema im Bildungsbereich vor allem in einer Krise nicht vernachlässigt werden sollte. Für die Gesundheitsförderung und Prävention sind vor allem ganzheitliche Strategien aussichtsreich, die sich an den gesundheitlichen Bedarfen aller schulischen Zielgruppen ausrichten (z. B. der Schüler*innen, des unterrichtenden und nicht unterrichtenden Schulpersonals), an verschiedenen Ebenen innerhalb der Schule (z. B. Unterricht, Schulorganisation, Schulumwelt) sowie außerhalb der Schule (z. B. Kooperation mit außerschulischen Akteuren) ansetzen und den Leitprinzipien der Partizipation und Vernetzung folgen [16]. Im Gegensatz zu Maßnahmen, die ausschließlich auf das Gesundheitsverhalten zielen, zeichnen sich settingbezogene Ansätze der gesundheitsfördernden Schule (engl. Health Promoting School) durch eine Verknüpfung von verhaltens- und verhältnispräventiven Maßnahmen aus, weshalb diese als geeignete Strategie zur Reduktion des Präventionsdilemmas (d. h. eingeschränkte Erreichbarkeit von Gruppen mit dem höchsten Präventionsbedarf) gesehen werden [16, 17]. Auch international gelten ganzheitliche Ansätze („whole school approaches“) als favorisierte Konzepte der schulischen Gesundheitsförderung [18, 19]. Entsprechende Evaluationsbefunde weisen auf eine Stärkung gesundheitsförderlicher Verhaltensweisen (z. B. körperliche Aktivität, Obst- und Gemüsekonsum), eine Verbesserung des Schulklimas, des Body-Mass-Index sowie verschiedener Indikatoren der psychischen Gesundheit [20–23].

Angesichts der eingeschränkten Studienlage zur Implementation bleibt offen, ob und in welchem Ausmaß Schulen gesundheitsförderliche und präventive Maßnahmen in pandemischen Krisenzeiten umsetzen. Hierzu liegen bislang weder national noch international Erkenntnisse vor. Lediglich eine Studie aus Norditalien hat sich mit der Sichtweise von schulischen Leitungspersonen bezüglich des Ansatzes der gesundheitsfördernden Schule im Kontext der COVID-19-Pandemie beschäftigt [24]. Die Ergebnisse verdeutlichen die Vorteile, die ein ganzheitlicher Ansatz aus Sicht der Befragten hat. Dabei wurden verschiedene Aktivitäten im Kontext der COVID-19-Pandemie als wichtig erachtet, die sich den einzelnen Dimensionen der gesundheitsfördernden Schule zuordnen ließen (z. B. *individuelle Kompetenzen*: Gesundheitskompetenz, Bewältigungsfähigkeit; *soziale Schulumwelt*: Führungsstil, Gefühl der Zugehörigkeit; *kommunale Zusammenarbeit*: intersektorale Aktivitäten, Fortbildungen).

Vor dem Hintergrund begrenzter empirischer Erkenntnisse beschäftigt sich die vorliegende Studie mit der Frage, in welchem Ausmaß Schulen in Deutschland während der COVID-19-Pandemie Maßnahmen der Gesundheitsförderung und Prävention umsetzen. Dabei werden mögliche Unterschiede der Umsetzung nach Alter und Geschlecht der Schulleitung, Schulform und Bundesland ermittelt. Zudem soll der Frage nachgegangen werden, ob die Teilnahme an größeren Landesinitiativen bzw. der Einsatz landesweiter Präventionskonzepte mit einem besseren Umsetzungstand der schulischen Gesundheitsförderung während der COVID-19-Pandemie einhergeht.

## Methodik

### Studiendesign und Stichprobe

Die Studie ist Teil des internationalen COVID-Health-Literacy(COVID-HL)-Forschungsnetzwerks (www.covid-hl.org), das sich seit seiner Initiierung im April 2020 der Umsetzung empirischer Forschung im Bereich Gesundheitskompetenz, Gesundheitsinformation, Gesundheitsförderung und Prävention sowie der Gesundheit unterschiedlicher Bevölkerungsgruppen und Settings widmet. Mittlerweile gehören dem Netzwerk mehr als 140 Wissenschaftler*innen aus 64 Ländern an. Der COVID-HL-Schulleitungssurvey stellt nach einer Studierendenbefragung die zweite Studie des Netzwerks dar, die seit Februar 2021 in etwa 20 Ländern durchgeführt wird.

Zielgruppe der deutschen Untersuchung waren Schulleitungen sowie Mitglieder des Schulleitungsteams (z. B. stellvertretende Schulleitung) allgemeinbildender Schulen der Bundesländer Baden-Württemberg, Hessen, Niedersachsen und Nordrhein-Westfalen.

Die Durchführung der Studie erfolgte in Kooperation mit den jeweiligen Schulleitungsverbänden in Form einer Onlinebefragung vom 09.03. bis zum 13.04.2021, also im Zeitraum der dritten Infektionswelle in Deutschland. Hierfür wurden alle Schulleitungen über die E‑Mail-Verteiler der Verbände angeschrieben und zur Teilnahme an der Studie eingeladen. Zur Erhöhung der Rücklaufquote wurde nach etwa 10 Tagen eine Erinnerung versendet. Neben Informationen zu den Zielen, Inhalten und dem Datenschutz enthielt die Einladung einen Link zur Befragung. Die Teilnahme an der Befragung war freiwillig und konnte zu jedem Zeitpunkt abgebrochen werden. Die mittlere Bearbeitungszeit des Fragebogens betrug etwa 20 min.

### Instrument

Der *Umsetzungsstand der schulischen Gesundheitsförderung* wurde mithilfe einer eigenentwickelten Skala erfasst, welche an bestehende Vorarbeiten anknüpft [25]. Bei der Itementwicklung wurde darauf geachtet, dass die 3 Kerndimensionen ganzheitlicher Ansätze der schulischen Gesundheitsförderung (Curriculum, Schulumwelt, außerschulische Kooperation/Zusammenarbeit) abgebildet werden und damit neben Schüler*innen weitere schulische und außerschulische Personengruppen (z. B. Lehrkräfte, Eltern) Berücksichtigung finden.

Die Entwicklung erfolgte in verschiedenen Iterationen, die im Forschungsteam mehrfach diskursiv überarbeitet wurden. Die im Survey eingesetzte Version des Instruments umfasste 15 Items, die entlang einer vierstufigen Antwortskala hinsichtlich ihrer Zustimmung beantwortet werden konnten (1 = trifft überhaupt nicht zu, 2 = trifft eher nicht zu, 3 = trifft eher zu, 4 = trifft völlig zu). Ein exemplarisches Item lautet: „An unserer Schule lernen Schüler*innen, wie man sich trotz der Einschränkungen aufgrund des Coronavirus ausreichend bewegen kann.“

Die *Teilnahme an Landesinitiativen und Programmen der schulischen Gesundheitsförderung* wurde pro Bundesland mittels eines Items abgebildet. Die Beantwortung erfolgte für 3 der 4 Bundesländer über eine 5‑stufige Antwortskala (1 = nein, 2 = weniger als 1 Jahr, 3 = seit 1 bis < 2 Jahren, 4 = seit 2 bis < 3 Jahren, 4 = seit 3 Jahren und länger), wobei für die bivariaten Analysen die Antworten dichotomisiert wurden (keine Teilnahme versus Teilnahme). Im Fokus standen jeweils landesweit implementierte Programme mit intersektoraler Akteursstruktur.

Befragte aus Nordrhein-Westfalen wurden nach der Teilnahme am Landesprogramm „Bildung und Gesundheit“ (BuG) gefragt, an dem derzeit 342 Schulen mit etwa 270.000 Schüler*innen und 20.000 Lehrkräften teilnehmen. Während für Baden-Württemberg die Umsetzung des vom Ministerium für Kultus, Jugend und Sport getragenen Präventionskonzepts „stark.stärker.WIR“ (ssW) erfasst wurde, erfolgte für Niedersachsen die Ausrichtung am Programm „GESUND LEBEN LERNEN“ (GLL), an dem sich bislang 270 Schulen beteiligt haben. Im Gegensatz zu den anderen Bundesländern besteht in Hessen kein landesweites Programm, sondern die Möglichkeit der Zertifizierung als „gesundheitsfördernde Schule“. Befragte wurden hier um eine Angabe gebeten, ob die jeweilige Schule über ein solches Teil- oder Gesamtzertifikat verfügt.

Als *demografische und Schulmerkmale* wurden das Geschlecht (männlich, weiblich, divers) und das Alter (≤ 45 Jahre, 46–50 Jahre, 51–55 Jahre, 56–60 Jahre, > 60 Jahre) der befragten Leitungspersonen, die Schulform und das Bundesland erfasst. Da sich die Schulformen zwischen den Bundesländern unterscheiden, wird im Folgenden lediglich eine Einteilung in Grund- und Sekundarschulen vorgenommen. Personen mit Geschlechtsangabe „divers“ wurden aufgrund der geringen Fallzahl (*n* = 1) in den bivariaten Geschlechtsanalysen nicht berücksichtigt.

### Statistische Analysen

Da die Erfassung des Umsetzungsstands der schulischen Gesundheitsförderung anhand neu entwickelter Items erfolgte, wurde in einem ersten Schritt die korrelative Struktur mittels Hauptkomponentenanalyse (PCA) und anschließender orthogonaler Varimax-Rotation überprüft. Hierfür wurden in einem initialen Durchgang alle 15 Items eingeschlossen und die Eignung der Items anhand des Kaiser-Meyer-Olkin-Kriteriums (KMO > 0,6) und des Bartlett-Tests auf Sphärizität (*p* < 0,05) untersucht [26, 27]. Die Extraktion der Items erfolgte unter Zuhilfenahme des Scree-Tests mit Eigenwerten > 1. Anschließend wurden Items mit geringen Kommunalitäten (< 0,45) und Faktorladungen (< 0,5) entfernt, wobei die Items vor Ausschluss auch hinsichtlich ihrer inhaltlichen Relevanz bewertet wurden [26, 27]. Mithilfe der verbliebenen Items wurde eine erneute PCA berechnet und anhand ihrer statistischen Güte final bewertet. Die Zuverlässigkeit der extrahierten Items aus der initialen und finalen PCA wurde anhand ihrer internen Konsistenz (Cronbachs Alpha) beurteilt.

Anschließend erfolgte eine univariate Auswertung der Umsetzung der schulischen Gesundheitsförderung anhand von relativen Häufigkeiten. Die Analyse von Unterschieden des Umsetzungstandes der schulischen Gesundheitsförderung nach demografischen Faktoren (Geschlecht, Alter), Schulform und Bundesland erfolgte mithilfe von Kreuztabellen mit angeschlossenem Chi-Quadrat-Test (χ^2^). Hierfür wurde eine Dichotomisierung der Skalenmittelwerte der in der PCA ermittelten Faktoren anhand des Median-Splits vorgenommen (niedriger Umsetzungsstand (untere 50 %) und hoher Umsetzungsstand (obere 50 %)).

Analog wurde im Hinblick auf die Stratifizierung nach Teilnahme an größeren Landesinitiativen und -programmen vorgegangen. Aufgrund zum Teil geringer Fallzahlen für die Teilnahme an Landesprogrammen wurde in den bivariaten Analysen lediglich eine Unterscheidung dahin gehend vorgenommen, ob ein Zertifikat vorlag bzw. an einem Landesprogramm (BuG, GLL, ssW) teilgenommen wurde. Für die χ^2^-Unabhängigkeitstests wurde ein Signifikanzniveau von *p* < 0,050 festgelegt. Alle Berechnungen wurden mit der Statistiksoftware IBM SPSS Statistics 25 (IBM Corp., Armonk, NY, USA) vorgenommen.

## Ergebnisse

### Beschreibung der Stichprobe

Nach Datenbereinigung umfasst die finale Stichprobe 2186 Schulleitungen und Mitglieder der Schulleitungsteams (z. B. stellvertretende Schulleitungen). Dabei überwiegen weibliche Schulleitungen mit 66,1 % und Grundschulleitungen geringfügig mit 53,2 % (Tab. 1). Etwa 45 % der Befragten gehören in die Alterskategorie der bis 50-Jährigen, während 12,6 % älter als 60 Jahre sind. Mit Blick auf das Bundesland kommt etwa ein Drittel der Befragten aus Hessen und jeweils etwa 25 % aus Niedersachsen und Nordrhein-Westfalen. Schulleitungen aus Baden-Württemberg nehmen einen Anteil von etwa 16 % an der Gesamtstichprobe ein. In Ermangelung öffentlich verfügbarer Daten sind Vergleiche mit der Grundgesamtheit von Schulleitungen und Schulleitungsmitgliedern der entsprechenden Bundesländer und damit Aussagen zu möglichen Verzerrungen nicht möglich.

**Table 1:** 

	Häufigkeit (*n*)	Anteil (%)
*Geschlecht*		
Männlich	739	33,8
Weiblich	1446	66,1
Divers	1	0,1
*Alter*		
≤ 45 Jahre	476	21,8
46 bis 50	508	23,3
51 bis 55	500	22,9
56 bis 60	424	19,4
> 60 Jahre	275	12,6
*Schulform*		
Grundschule	1138	53,2
Sekundarschule	1003	46,8
*Bundesland*		
Baden-Württemberg	338	15,5
Hessen	713	32,6
Niedersachsen	572	26,2
Nordrhein-Westfalen	564	25,8
*Gesamt*	*2186*	*100,0*

### Faktorielle Struktur der COVID-19-bezogenen schulischen Gesundheitsförderung

Die 15 Items zur Erfassung der COVID-19-bezogenen schulischen Gesundheitsförderung erweisen sich unter Rückgriff auf die statistischen Gütekriterien für eine PCA als geeignet (KMO = 0,89; Bartlett-Test = χ^2^ = 8,942; *p* < 0,001). Auf Basis der PCA ließen sich 3 Faktoren mit einem Eigenwert > 1 extrahieren, die insgesamt 50,9 % der Gesamtvarianz erklären (Onlinematerial Tab. Z1). Für Faktor 1 liegen die Ladungen zwischen 0,58 und 0,77, während die Ladungen des zweiten Faktors zwischen 0,44 und 0,67 und die des dritten Faktors zwischen 0,65 und 0,87 rangieren.

Insgesamt umfasst Faktor 1 5 Items, die sich mit wissens- und verhaltensbezogenen Aspekten der Gesundheit von Schüler*innen im Kontext der COVID-19-Pandemie beschäftigen (z. B. „An unserer Schule lernen Schüler*innen, wie man sich trotz der Einschränkungen aufgrund des Coronavirus ausreichend bewegen kann“). Hingegen beschreiben die 7 Items von Faktor 2 übergreifende Prinzipien der schulischen Gesundheitsförderung wie die Kooperation und Partizipation (z. B. „An unserer Schule arbeiten wir eng mit den Eltern zusammen, wenn es um die Förderung und den Schutz der Gesundheit der Kinder geht“). Schließlich vereint der dritte Faktor 3 Items, die vor allem die gesundheitsförderliche Gestaltung von Lehr‑, Lern- und Arbeitsbedingungen adressieren und damit Aspekte der Lehrkräftegesundheit aufgreifen (z. B. „An unserer Schule wird das Schulpersonal im Umgang mit Belastungssituationen durch das Coronavirus [z. B. Stress] unterstützt“).

Da 3 Items die vorab definierten Kriterien nicht erfüllten und ein Item auf 2 Faktoren in ähnlicher Größe lädt, wurden diese entfernt. Die Korrelationsmatrix der verbliebenen 12 Items erweist sich ebenfalls für eine PCA als geeignet (KMO = 0,87; Bartlett-Test = χ^2^ = 7,413; *p* < 0,001). Die Ergebnisse des Scree-Tests ergeben ebenfalls 3 Faktoren, die zusammen 57 % der Gesamtvarianz erklären (siehe zusammenfassend Tab. 2).

**Table 2:** 

Item Nr.	Beschreibung: An unserer Schule …	Faktor 1(α = 0,76)	Faktor 2(α = 0,81)	Faktor 3(α = 0,65)
Item 2	… lernen Schüler*innen Möglichkeiten, wie man sich vor einer Ansteckung schützen kann	*0,771*	0,058	0,105
Item 1	… werden den Schüler*innen grundlegende Informationen über das Coronavirus vermittelt (z. B. Ursachen der Entstehung, Verbreitung)	*0,715*	0,046	0,096
Item 3	… lernen Schüler*innen, wie man sich trotz der Einschränkungen aufgrund des Coronavirus ausreichend bewegen kann	*0,705*	0,244	0,228
Item 5	… werden Schüler*innen im Umgang mit Sorgen und Ängsten durch das Coronavirus unterstützt	*0,580*	0,297	0,230
Item 4	… lernen Schüler*innen, wie man sich trotz der Einschränkungen aufgrund des Coronavirus gesund ernähren kann	*0,560*	0,246	0,260
Item 8	… spielen gesundheitsförderliche Aspekte bei der Gestaltung der Arbeitsbedingungen (einschließlich Homeoffice) eine wichtige Rolle	0,104	*0,879*	0,192
Item 7	… spielen gesundheitsförderliche Aspekte bei der Gestaltung der Unterrichts- und Lernbedingungen (einschließlich Homeschooling) eine wichtige Rolle	0,170	*0,863*	0,210
Item 6	… wird das Schulpersonal im Umgang mit Belastungssituationen durch das Coronavirus (z. B. Stress) unterstützt	0,292	*0,657*	0,177
Item 12	… arbeiten wir eng mit kommunalen Akteuren aus dem Bereich Gesundheit und Soziales zusammen, wenn es um die Förderung und den Schutz der Gesundheit der Schüler*innen geht	0,108	0,088	*0,733*
Item 11	… arbeiten wir eng mit den Eltern zusammen, wenn es um die Förderung und den Schutz der Gesundheit der Kinder geht	0,212	0,144	*0,669*
Item 14	… werden Schüler*innen bei der Planung von Maßnahmen der Prävention und Gesundheitsförderung einbezogen	0,130	0,152	*0,635*
Item 13	… besteht Konsens darüber, dass Gesundheit und Schulleistung der Schüler*innen zusammenhängen	0,230	0,275	*0,589*
*Erklärte Varianz (gesamt 57,0* *%)*		*37,6* *%*	*10,7* *%*	*8,7* *%*

*α* Cronbachs Alpha, *Faktor 1* COVID-19-bezogene Unterstützung der Schüler*innen, *Faktor 2* Gesundheitsförderliche Lehr‑, Lern- und Arbeitsbedingungen, *Faktor 3* Prinzipien der Gesundheitsfördernden Schule

Insgesamt umfassen die Faktoren 3 Items (Faktor 2), 4 Items (Faktor 3) und 5 Items (Faktor 1) mit Faktorladungen zwischen 0,56 bis 0,88. Die Ergebnisse der finalen Lösung lassen sich analog der initialen PCA interpretieren: „COVID-19-bezogene Unterstützung der Schüler*innen“ (Faktor 1), „Gesundheitsförderliche Lehr‑, Lern- und Arbeitsbedingungen“ (Faktor 2) und „Prinzipien der Gesundheitsfördernden Schule“ (Faktor 3). Die 3 finalen Faktoren korrelieren auf mittlerem Niveau (*r* = 0,48–0,51) und erreichen zufriedenstellende bis gute Reliabilitätswerte (0,65 < α < 0,81; Onlinematerial Tab. Z1).

### Umsetzung der COVID-19-bezogenen schulischen Gesundheitsförderung

Im Vergleich der 3 ermittelten Faktoren findet sich der höchste Umsetzungsstand für COVID-19-bezogene Unterstützung der Schüler*innen (Faktor 1). Über alle Items hinweg liegt der prozentuale Anteil mit geringer Umsetzung (Antwortoption: trifft eher nicht oder überhaupt nicht zu) bei 13 %. Die höchste Zustimmung (98,9 %) ergibt sich für die Vermittlung von Möglichkeiten, wie man sich vor einer Ansteckung schützen kann, während fast 32 % der Befragten der Vermittlung von Möglichkeiten zur gesunden Ernährung weniger Aufmerksamkeit widmen.

Der Anteil der Befragten, die eine geringe Umsetzung der *Prinzipien der Gesundheitsfördernden Schule* (Faktor 3) berichten, beträgt über alle Items hinweg 28,9 %. Dabei finden sich die höchsten Zustimmungswerte dahin gehend, dass an der Schule ein Konsens über den Zusammenhang von Gesundheit und Schulleistung besteht (93,3 %). Hingegen geben mehr als die Hälfte der Befragten (52 %) an, dass Schüler*innen bei der Planung von Maßnahmen der Gesundheitsförderung und Prävention eher nicht oder überhaupt nicht einbezogen werden. 42 % geben keine enge Zusammenarbeit mit kommunalen Akteur*innen zu Fragen der Schüler*innengesundheit an.

Für den Faktor 2 *„Gesundheitsförderliche Lehr‑, Lern- und Arbeitsbedingungen“* lässt sich über alle 3 Items für 31,2 % ein geringer Umsetzungsstand feststellen (Antwortoption: trifft eher nicht oder überhaupt nicht zu), wobei die Unterschiede zwischen den Items maximal 5 % betragen. Dabei stimmen 71,5 % der Aussage eher oder völlig zu, das Schulpersonal im Umgang mit Belastungssituationen durch das Coronavirus zu unterstützen. Für die gesundheitsförderliche Gestaltung der Arbeitsbedingungen (einschließlich des Homeoffice) ergibt sich mit 66,5 % Zustimmung ebenfalls ein hoher Umsetzungsstand.

### Unterschiede der COVID-19-bezogenen schulischen Gesundheitsförderung nach demografischen Faktoren, Schulform und Bundesland

Unter Berücksichtigung der Faktoren Geschlecht, Alter, Schulform und Bundesland ergeben sich in den bivariaten Analysen signifikante Unterschiede im Umsetzungsstand der schulischen Gesundheitsförderung (Tab. 3). Während weibliche Schulleitungen eine höhere Umsetzung von COVID-19-bezogenen Unterstützungsmaßnahmen (χ^2^(1) = 11,80; *p* < 0,010) und Prinzipien der Gesundheitsfördernden Schule (χ^2^(1) = 9,97; *p* < 0,001) berichten, findet sich bei Befragten > 60 Jahre ein höherer Umsetzungsstand für Aspekte der gesundheitsförderlichen Lehr‑, Lern- und Arbeitsbedingungen (χ^2^(4) = 11,67; *p* < 0,050). Hingegen berichten Grundschulleitungen im Vergleich zu Befragten aus weiterführenden Schulen einen höheren Implementierungsgrad von Prinzipien der Gesundheitsfördernden Schule (χ^2^(1) = 7,14; *p* < 0,010). Schließlich ließen sich signifikante Unterschiede in allen 3 Faktoren der schulischen Gesundheitsförderung zugunsten von Schulen aus Niedersachsen (COVID-19-bezogene Unterstützung von Schüler*innen), Nordrhein-Westfalen (gesundheitsförderliche Lehr‑, Lern- und Arbeitsbedingungen) und Baden-Württemberg (Prinzipien der Gesundheitsfördernden Schule) feststellen (*p* < 0,050 bis* p* < 0,0010).

**Table 3:** 

	Faktor 1: COVID-19-bezogene Unterstützung der SuS		Faktor 2: Lehr‑, Lern- und Arbeitsbedingungen		Faktor 3: Prinzipien der Gesundheitsfördernden Schule	
	Niedrig	Hoch	Niedrig	Hoch	Niedrig	Hoch
	% (*n*)	% (*n*)	% (*n*)	% (*n*)	% (*n*)	% (*n*)
**Geschlecht**	*χ2 (df* *=* *1)* *=* *11,80, p* *<* *0,01*		n. s.		*χ2 (df* *=* *1)* *=* *9,967, p* *<* *0,001*	
Männlich	58,9 % (402)	41,1 % (281)	72,8 % (501)	27,2 % (187)	73,7 % (502)	26,3 % (179)
Weiblich	50,8 % (687)	49,2 % (665)	75,5 % (1025)	24,5 % (332)	66,9 % (904)	33,1 % (448)
**Alter**	n. s.		*χ2 (df* *=* *4)* *=* *11,671, p* *<* *0,05*		n. s.	
≤ 45 Jahre	58,0 % (258)	42,0 % (187)	77,6 % (343)	22,4 % (99)	70,1 % (310)	29,9 % (132)
46 bis 50	51,6 % (246)	48,4 % (231)	77,4 % (369)	22,6 % (108)	69,5 % (330)	30,5 % (145)
51 bis 55	53,2 % (247)	46,8 % (217)	73,0 % (340)	27,0 % (126)	67,4 % (312)	32,6 % (151)
56 bis 60	50,9 % (200)	49,1 % (193)	74,8 % (299)	25,3 % (101)	69,8 % (278)	30,2 % (120)
> 60 Jahre	53,9 % (137)	46,1 % (117)	67,4 % (174)	32,6 % (84)	69,6 % (176)	30,4 % (77)
**Schulform**	*n.* *s.*		*n.* *s.*		*χ2 (df* *=* *1)* *=* *7,137, p* *<* *0,01*	
Grundschule	51,6 % (551)	48,4 % (517)	74,3 % (794)	25,7 % (275)	66,3 % (708)	33,7 % (360)
Sekundarschule	55,7 % (516)	44,3 % (411)	75,0 % (701)	25,0 % (234)	71,8 % (666)	28,2 % (261)
**Bundesland**	*χ2 (df* *=* *3)* *=* *11,913, p* *<* *0,01*		*χ2 (df* *=* *3)* *=* *16,938, p* *<* *0,001*		*χ2 (df* *=* *3)* *=* *7,752, p* *<* *0,05*	
Baden-Württemberg	58,3 % (182)	41,7 % (130)	79,3 % (245)	20,7 % (64)	65,0 % (202)	35,0 % (109)
Hessen	56,9 % (381)	43,1 % (289)	77,4 % (521)	22,6 % (152)	70,8 % (471)	29,2 % (194)
Niedersachsen	48,8 % (258)	51,2 % (271)	74,4 % (396)	25,6 % (136)	72,2 % (385)	27,8 % (148)
Nordrhein-Westfalen	51,1 % (269)	48,9 % (257)	68,5 % (365)	31,5 % (168)	66,3 % (202)	33,7 % (177)

Einteilung in niedrige und hohe Umsetzung der schulischen Gesundheitsförderung anhand des Medians (untere und obere 50 % der Angaben in der Stichprobe)

*n* Häufigkeit, χ^2^ Chi-Quadrat, *n.* *s.* nicht signifikant, *SuS* Schülerinnen und Schüler

### Unterschiede der COVID-19-bezogenen schulischen Gesundheitsförderung nach Teilnahme an einem Landesprogramm

Mehr als ein Drittel (38,8 %) der hessischen Befragten berichtet, dass ihre Schule über ein Teil- oder Vollzertifikat im Bereich Schule und Gesundheit verfügt (davon 9,4 % mit Vollzertifikat). Aus den anderen in die Studie eingeschlossenen Bundesländern berichten 14,1 % der Schulleitungen bzw. Schulleitungsmitglieder an einem Landesprogramm zur schulischen Gesundheitsförderung teilzunehmen bzw. nach einem Landeskonzept zu arbeiten. Differenziert nach Bundesland finden sich die höchsten Teilnahmeraten für das Präventionskonzept „stark.stärker.WIR“ (25,1 % der Befragten aus Baden-Württemberg), während 13,3 % der Befragten aus Nordrhein-Westfalen angeben, am Landesprogramm „Bildung und Gesundheit“ teilzunehmen. Mit 8,2 % findet sich für Befragte aus Niedersachsen die geringste Teilnahmerate am Programm „GESUND LEBEN LERNEN“.

**Figure 1:**
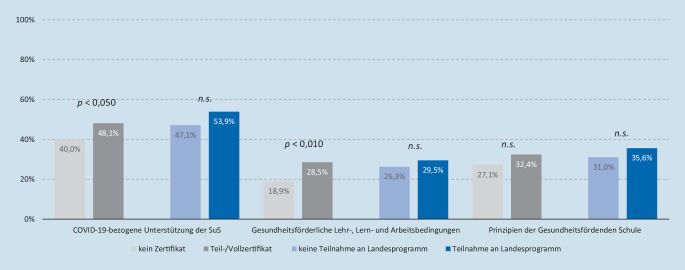

In den bivariaten Analysen zeigen sich lediglich für Befragte aus Hessen signifikante Unterschiede für 2 der 3 Dimensionen des Umsetzungsstands der schulischen Gesundheitsförderung. Schulleitungen, die über ein Teil- oder Vollzertifikat verfügen, weisen häufiger einen hohen Umsetzungsstand von COVID-19-bezogenen Unterstützungsmaßnahmen für Schüler*innen (χ^2^(1) = 4,23; *p* < 0,050) sowie der Gestaltung gesundheitsförderlicher Lehr‑, Lern- und Arbeitsbedingungen (χ^2^(1) = 8,367; *p* < 0,010) auf (Abb. 1). Hingegen ließ sich für Befragte, die an einem Landesprogramm oder -konzept teilnehmen, in keiner der Dimensionen der schulischen Gesundheitsförderung ein höherer Umsetzungsstand feststellen.

## Diskussion

Ziel der vorliegenden Arbeit war es, die Umsetzung von Maßnahmen der schulischen Gesundheitsförderung im Kontext der COVID-19-Pandemie zu untersuchen. Auf Basis des hierbei eingesetzten Instruments ließen sich 3 Dimensionen der schulischen Gesundheitsförderung empirisch abbilden: (1) COVID-19-bezogene Unterstützung von Schüler*innen, (2) Gestaltung gesundheitsförderlicher Lehr‑, Lern- und Arbeitsbedingungen und (3) Prinzipien der Gesundheitsfördernden Schule.

Dabei zeigen die univariaten Analysen, dass COVID-19-bezogene Unterstützungsmaßnahmen bei Schüler*innen aus Sicht von Schulleitungen am stärksten adressiert werden, wohingegen Aspekte der gesundheitsförderlichen Gestaltung von Lehr‑, Lern- und Arbeitsbedingungen eine deutlich geringere Umsetzung aufweisen. Dieser Befund deckt sich grundsätzlich mit den Erkenntnissen einer vor der COVID-19-Pandemie durchgeführten Serie von Studien mit Schulleitungen [25]. Auch hier ließ sich für die Förderung des Gesundheitsverhaltens der Schüler*innen der höchste Implementierungsstand feststellen.

Dies mag u. a. daran liegen, dass Schulen entsprechend der schulgesetzlichen Vorgaben einen Gesundheitsbildungsauftrag haben, der in den Bundesländern unterschiedlich verankert ist. So stellt die Gesundheitsbildung im Schulgesetz Hessen eine besondere Bildungs- und Erziehungsaufgabe dar, die als Aufgabengebiet fächerübergreifend unterrichtet wird (§ 6 Schulgesetz (SchulG) Hessen). Während im niedersächsischen Schulgesetz in § 2 festgehalten ist, dass die Schüler*innen befähigt werden sollen, „… für die Erhaltung der Umwelt Verantwortung zu tragen und gesundheitsbewusst zu leben“, sollen Schüler*innen laut § 2 SchulG Nordrhein-Westfalen lernen, „… Freude an der Bewegung und am gemeinsamen Sport zu entwickeln, sich gesund zu ernähren und gesund zu leben“. Mittlerweile liegen auf curricularer Ebene zahlreiche Materialien und Programme vor, die für die Vermittlung gesundheitsbezogener Themen im Unterricht eingesetzt werden können und zum Teil an den Landesrahmenlehrplänen ausgerichtet sind [28].

Hingegen sind Aspekte der Lehrergesundheit und der Gestaltung gesundheitsförderlicher Arbeitsbedingungen zwar im Arbeitsschutzgesetz (ArbSchG) verankert, z. B. § 5 Abs. 3 Nr. 6 im Rahmen der psychischen Gefährdungsbeurteilung, und in Nordrhein-Westfalen sogar im Schulgesetz (§ 59, Punkt 8), jedoch erfordern diese seitens der Schule und der Schulleitung mehr Aufwand, da sie in schulische Strukturen und Organisationsentwicklungsprozesse integriert sind [29]. Angesichts der COVID-19-bezogenen Arbeitsverdichtung mag dies ein Grund dafür sein, dass dem Belastungsgeschehen von Lehrkräften und der Gestaltung gesundheitsförderlicher Arbeitsbedingungen weniger Aufmerksamkeit zuteilwird.

Zu berücksichtigen ist dabei jedoch, dass die Lehrgesundheit eine zentrale Ressource für Schulqualität darstellt und mit Bildungsergebnissen von Schüler*innen assoziiert ist [30]. So zeigen aktuelle Befunde, dass die emotionale Erschöpfung bei Lehrkräften aus Sicht von Schüler*innen mit einer geringeren emotionalen Unterstützung sowie einer geringeren Unterrichtsorganisation verbunden ist [31], wobei die Unterrichtsqualität den Zusammenhang zwischen der Erschöpfung von Lehrkräften und den Bildungsergebnissen von Schüler*innen partiell mediiert.

Schließlich ergab sich mit Blick auf grundlegende Prinzipien der Gesundheitsfördernden Schule eine geringe Umsetzung für die Kooperation mit kommunalen Akteuren sowie eine geringe Beteiligung von Schüler*innen an der Planung von Maßnahmen der Gesundheitsförderung und Prävention. Vorliegende Übersichtsarbeiten weisen darauf hin, dass Partizipation im Sinne einer aktiven Mitbestimmung und -gestaltung mit positiven Interventionseffekten, u. a. auf individueller und organisationaler Ebene, verbunden ist [32, 33]. Ob das Ausmaß an Partizipation in der schulischen Gesundheitsförderung infolge der COVID-19-Pandemie (z. B. aufgrund der durch Hygienebestimmung reduzierten Möglichkeit des persönlichen Austauschs oder einer allgemein abnehmenden Priorität von Maßnahmen der Gesundheitsförderung) abgenommen hat, lässt sich aufgrund des Mangels an Vergleichsdaten nicht bestimmen. Jedoch sollten Schulen zukünftig verstärkt darin unterstützt werden, ihre Maßnahmen entsprechend ganzheitlicher Ansätze der schulischen Gesundheitsförderung partizipativ auszurichten und Schüler*innen wie auch andere Personengruppen in allen Phasen der Maßnahmenplanung und -umsetzung zu beteiligen.

Angesichts der in der Einleitung beschriebenen gesundheitlichen Bedarfe, die zum Teil bereits vor der COVID-19-Pandemie bestanden, nun aber an Bedeutung gewonnen haben, ist der systematische Ausbau von Kooperations- und Netzwerkstrukturen mit kommunalen Fachakteuren anzustreben. Hierfür liegen bereits gut evaluierte Rahmenmodelle [34] sowie erste digital gestützte Interventionen [35] vor, die von Schulen eingesetzt werden können.

Wie bereits in vorhergehenden Untersuchungen festgestellt [25], ließen sich in der vorliegenden Studie Unterschiede im Umsetzungsstand zugunsten von weiblichen und älteren Schulleitungen feststellen. Als mögliche Erklärung ließe sich anführen, dass Frauen für gesundheitliche Themen sensitiver sind und auch Präventionsangebote häufiger in Anspruch nehmen [36], während ältere Schulleitungen möglicherweise eine größere Arbeitserfahrung aufweisen und daher der Gesundheit im Kontext von Bildungsqualität und Arbeitsfähigkeit einen höheren Stellenwert beimessen.

Überraschend ist hingegen, dass sich mit Ausnahme des Faktors „Prinzipien der Gesundheitsfördernden Schule“ keine durchgehenden Schulformunterschiede zugunsten von Grundschulen nachweisen ließen. Gerade Grundschulen weisen gegenüber den Sekundarschulen eine stärkere Ausrichtung an der ganzheitlichen Entwicklung des Kindes auf, während sich Sekundarschulen durch eine stärkere Fächerorientierung auszeichnen. Ob die fehlenden Unterschiede auf die COVID-19-Pandemie (z. B. weniger Möglichkeiten der Umsetzung von Maßnahmen für Grundschulen durch Sicherstellung des Schulbetriebs) zurückgeführt werden können, muss weitergehend untersucht werden.

Schließlich weisen die Ergebnisse der vorliegenden Studie darauf hin, dass die Teilnahme an einem Landesprogramm bzw. die Ausrichtung an einem Landespräventionskonzept nicht mit einem höheren Umsetzungsstand der schulischen Gesundheitsförderung und Prävention einhergeht. Auch hier ist ein Vergleich in Ermangelung an Studienbefunden sehr eingeschränkt. Lediglich eine qualitative Studie von Matern und Stauf [37] hat Grundschulen, die am nordrhein-westfälischen Landesprogramm „Bildung und Gesundheit“ (BuG) teilnahmen, mit einer Vergleichsgruppe von nicht am Landesprogramm involvierten Schulen hinsichtlich der Implementation von Gesundheitsförderung verglichen. Dabei ergab die Sichtung aller Schulprogramme deutliche Unterschiede ernährungsbezogener Maßnahmen zugunsten der BuG-Schulen.

Vor diesem Hintergrund lässt sich die Vermutung anstellen, dass die an den Programmen BuG, GLL und ssW teilnehmenden Schulen aufgrund der COVID-19-Pandemie und der damit verbundenen Herausforderungen und fehlender Ressourcen (z. B. Zeit, Personal) ihre Aktivitäten der schulischen Gesundheitsförderung nicht in der vorgesehenen Form umsetzen konnten. Ob auch seitens der Anbieter dieser Landesprogramme und -initiativen infolge pandemiebedingter Anforderungen nicht die erforderliche Unterstützung zur Umsetzung gesundheitsförderlicher Maßnahmen geleistet werden konnte, bedarf weiterer Untersuchungen. Hierbei gilt es auch, genauer zu analysieren, welche Mechanismen für hessische Schulen mit Teil- oder Vollzertifikat einen höheren Umsetzungsstand erklären können.

### Stärken und Limitation

Mit der vorliegenden Studie werden erste Befunde zur Umsetzung der schulischen Gesundheitsförderung im Kontext der COVID-19-Pandemie vorgelegt, die sich als Referenzpunkt für künftige Untersuchungen in diesem Bereich eignen. Dabei erlaubt das Querschnittsdesign der Studie keine kausalen Rückschlüsse. Verzerrungen, z. B. infolge der hohen Beteiligung weiblicher Befragter sind vorstellbar, jedoch aufgrund fehlender öffentlich zugänglicher Daten zur Grundgesamtheit nicht überprüfbar.

Zur Erfassung des Umsetzungsstandes kam ein neu entwickeltes Instrument zum Einsatz, welches verschiedene Facetten ganzheitlicher Ansätze der schulischen Gesundheitsförderung erfasst. Während in der internationalen Diskussion 3 Hauptelemente ganzheitlicher Ansätze diskutiert werden (Gesundheitsförderung auf (1) curricularer Ebene, (2) Ebene der Schulumwelt/-struktur und (3) Ebene der Zusammenarbeit mit außerschulischen Partnern), sind im finalen Instrument der vorliegenden Studie Aspekte der (sozialen) Schulumwelt und -struktur nach Prüfung der faktoriellen Struktur nicht hinreichend abgebildet. Hieraus ergibt sich ein Bedarf zur Weiterentwicklung des eingesetzten Instruments.

Die im Rahmen der Analyse vorgenommene Dichotomisierung der Dimensionen des Umsetzungsstands von schulischer Gesundheitsförderung und Prävention kann mit einem Informationsverlust einhergehen. Schließlich ist bei der Interpretation zu berücksichtigen, dass aufgrund zum Teil geringer Fallzahlen die Teilnahme an einem Landesprogramm für Baden-Württemberg, Niedersachsen und Nordrhein-Westfalen zusammengefasst wurde, womit differenzierte Aussagen für die dahinterliegenden Programme und Präventionskonzepte nicht möglich sind.

### Fazit und Implikationen

Die COVID-19-Pandemie hat seit 2020 zahlreiche soziale, bildungsbezogene und gesundheitliche Konsequenzen nach sich gezogen, die für die Public-Health-Forschung und -Praxis von erheblicher Bedeutung sind. Mehr denn je stellt die Schule einen geeigneten Ort dar, junge Menschen frühzeitig und ungeachtet ihres sozioökonomischen und kulturellen Hintergrunds mit ganzheitlichen Maßnahmen der Gesundheitsförderung und Prävention zu adressieren. Die COVID-19-Pandemie kann als disruptives Ereignis verstanden werden, welches die Umsetzung komplexer Interventionsansätze schulischer Gesundheitsförderung (z. B. infolge von Schulschließungen) deutlich erschwert.

Mögliche Ansatzpunkte für zukünftige Aktivitäten umfassen: (1) die Etablierung eines kontinuierlichen Monitorings zur Implementierung der schulischen Gesundheitsförderung in Deutschland unter Berücksichtigung der schulischen Bedarfe, (2) die systematische Unterstützung und Befähigung von Schulleitungen als „Gatekeeper“ gesundheitsförderlicher Entwicklungsprozesse an Schulen sowie (3) die Sicherstellung, dass landesbezogene Programme und Initiativen auch und vor allem in Krisensituationen Schulen mit ausreichend Ressourcen im Bereich Gesundheitsförderung und Prävention unterstützen können.

Großes Potenzial für die zielgerichtete Planung, Umsetzung und Evaluation von Maßnahmen der Gesundheitsförderung und -prävention bietet dabei das international bewährte Konzept der Schulgesundheitspflege, zu dem mittlerweile auch in Deutschland Erfahrung aus Modellvorhaben vorliegen [38]. Schließlich ist vor dem Hintergrund der bestehenden Public-Health-Herausforderungen (4) eine Neubestimmung der gesetzlichen Verankerung von schulischer Gesundheitsförderung und Prävention in der Schule vorzunehmen.

## Supplementary Information




