# Supplementary material for: School health promotion in pandemic times. Results of the COVID-HL school principal study
Source: Bundesgesundheitsblatt Gesundheitsforschung Gesundheitsschutz. 2022 May 6;65(7-8):758–67. [Article in German] doi: 10.1007/s00103-022-03535-w (PMC9073822; doi:10.1007/s00103-022-03535-w)
Supplement: Supplementary file 1 [file 103_2022_3535_MOESM1_ESM.pdf]

Bundesgesundheitsblatt – Gesundheitsforschung – Gesundheitsschutz, Ausgabe 7/2022

Onlinematerial zum Beitrag:

Schulische Gesundheitsförderung in pandemischen Zeiten. Ergebnisse der COVID-HL Schulleitungsstudie

Kevin Dadaczynski[1,2,3], Orkan Okan[4], Melanie Messer[5]

[1] Fachbereich Pflege und Gesundheit, Hochschule Fulda, Fulda, Deutschland
[2] Public Health Zentrum (PHZF), Hochschule Fulda, Fulda, Deutschland
[3] Zentrum für Angewandte Gesundheitswissenschaften, Leuphana Universität Lüneburg, Lüneburg, Deutschland
[4] Fakultät für Sport- und Gesundheitswissenschaften, Technische Universität München, München, Deutschland
[5] Abteilung Pflegewissenschaft II, Universität Trier, Deutschland

Korrespondenzadresse:

Prof. Dr. Kevin Dadaczynski
Hochschule Fulda
Fachbereich für Pflege und Gesundheit
Leipziger Straße 123
36037 Fulda
Deutschland
kevin.dadaczynski@pg.hs-fulda.de

Inhalt:

Tabelle Z1: Initiale und finale Hauptkomponentenlösung der COVID-19-bezogenen schulischen Gesundheitsförderung

Tabelle Z1: Initiale und finale Hauptkomponentenlösung der COVID-19-bezogenen schulischen Gesundheitsförderung

		Initiale 15-Item-Lösung	Finale 12-Item-Lösung
KMO		0,89	0,87
Bartlett-Test		$\chi^2 = 8{,}942$, $p<0{,}001$	$\chi^2 = 7{,}413$, $p<0{,}001$
Faktor Interkorrelation		0,48-0,55 ($p<0{,}001$)	0,48-0,51 ($p<0{,}001$)
Items	Faktor 1	1, 2, 3, 4, 5	1, 2, 3, 4, 5
	Faktor 2	9, 10, 11, 12, 13, 14, 15	6, 7, 8
	Faktor 3	6, 7, 8	11, 12, 13, 14
Eigenwerte	Faktor 1	5,206	4,510
	Faktor 2	1,330	1,285
	Faktor 3	1,091	1,039
Erklärte Varianz	Faktor 1	34,7%	37,6%
	Faktor 2	8,9%	10,7%
	Faktor 3	7,3%	8,7%
Kommunalitäten (> 0,40)	Faktor 1<br>Faktor 2<br>Faktor 3	0,36 (Item 9) -<br>0,80 (Item 8)	0,44 (Item 4) -<br>0,82 (Item 8)
Faktorladungen (> 0,50)	Faktor 1	0,575-0,768	0,560-0,773
	Faktor 2	0,441-0,665	0,657-0,879
	Faktor 3	0,649-0,868	0,589-0,733
Reliabilität (Cronbach α)	Faktor 1	0,76	0,76
	Faktor 2	0,73	0,81
	Faktor 3	0,81	0,65
